# Optimizing water–nitrogen coupling for quinoa yield, quality, and resource use efficiency in arid regions

**DOI:** 10.3389/fpls.2025.1654704

**Published:** 2025-12-19

**Authors:** Xinyue Li, Zhaotong Shen, Yingge A, Yupeng Zhao, Bin Yang, Yaozu Feng, Qingyong Bian, Yanbo Fu

**Affiliations:** 1 Institute of Microbiology, Xinjiang Uygur Autonomous Region Academy of Agricultural Sciences, Urumqi, China; 2 College of Resources and Environment, Xinjiang Agricultural University, Urumqi, China; 3 Institute of Agricultural Resources and Environment, Xinjiang Uygur Autonomous Region Academy of Agricultural Sciences, Urumqi, China; 4 National Soil Quality Aksu Observation Experimental Station, Xinjiang Academy of Agricultural Sciences, Aksu/National Soil Quality Observation and Experimental Station in Aksu Xinjiang, Aksu, China

**Keywords:** quinoa, water and fertilizer integration, yield, quality, water and nitrogen use efficiency

## Abstract

**Introduction:**

To investigate the effects of water–nitrogen coupling under drip irrigation on quinoa yield, quality, and water–nitrogen use efficiency, a microplot experiment was conducted in Aksu Prefecture, Xinjiang.

**Methods:**

The study employed a two-factor factorial design with three irrigation levels (*W*1: 2,100 m^3^/ha, *W*2: 2,700 m^3^/ha, *W*3: 3,300 m^3^/ha) and five nitrogen application rates (*N*0: 0 kg/ha, *N*1: 100 kg/ha, *N*2: 125 kg/ha, *N*3: 150 kg/ha, *N*4: 175 kg/ha), resulting in 15 treatment combinations.

**Results and discussion:**

Results indicated that under the *W*1 irrigation level, increasing nitrogen application enhanced dry matter accumulation. However, under *W*2 and *W*3 conditions, dry matter declined when nitrogen exceeded 150 kg/ha, indicating a significant nitrogen threshold effect. A yield response model (*R*² = 0.945) predicted a maximum theoretical yield of 4,363.91 kg/ha at an irrigation volume of 2,931.59 m^3^/ha and a nitrogen application rate of 149.24 kg/ha. Optimal grain quality—characterized by fat content of 51.47–55.90 g/kg, protein content of 189.68–199.05 g/kg, and starch content of 581.23–585.96 g/kg—was observed under *W*2 and *W*3 combined with *N*2 and *N*3 treatments. Regression analyses indicated peak values for water use efficiency (WUE), nitrogen agronomic efficiency (NAE), and nitrogen partial factor productivity (NPFP) at 1.64, 29.72, and 31.92 kg/kg, respectively. Based on these findings, a recommended water–nitrogen management strategy for quinoa production in southern Xinjiang is 2,700 m^3^/ha of drip irrigation water combined with 150 kg/ha of nitrogen fertilizer, achieving a balance among high yield, resource efficiency, and environmental sustainability.

## 1 Introduction

Water and nitrogen are not only important environmental factors for crop growth and development but also important components in determining crop yield (Song et al., 2024). Studies have shown that crops have significant differences in water and nitrogen requirements at different growth stages, and deficits during critical periods can significantly constrain the potential for yield increases, whereas excess water and nitrogen can result in resource wastage and pose ecological and environmental risks (Ma et al., 2024). Nitrogen fertilizer and water resources are important factors affecting agricultural production (Tang et al., 2024). China’s nitrogen fertilizer application is the highest in the world, but the average utilization rate is less than 35% (Feroz et al., 2024), and unabsorbed nitrogen causes problems such as groundwater pollution and soil acidification through leaching. Xinjiang is located in the crop growing season effective precipitation is small, the contradiction between water supply and demand and crop production contradictions are increasingly prominent, water and fertilizer integration technology through the precise control of water and nitrogen supply spatial and temporal coupling, can effectively reduce the loss of water and fertilizer in the transportation process, timely and sufficient to meet the crop in different periods of water and fertilizer demand, to promote synchronization of water and fertilizer in time and space, coupling, to achieve water-saving, fertilizer-saving, yield increase, high efficiency (Sameerkumar et al., 2016; Rathore et al., 2017).

Quinoa (*Chenopodium quinoa* Willd), also known as South American quinoa, is an annual herbaceous crop belonging to the genus *Chenopodium*, subfamily Chenopodioideae, of the family Amaranthaceae. Native to the Andean region, quinoa has been cultivated for more than 7,000 years (Kurenkova et al., 2021). During this growth, water and nitrogen are important factors affecting quinoa’s growth, yield, and quality, and reasonable water and nitrogen management is essential to ensure high yield and quality. Nitrogen is the main source of organic substances such as protein and fat produced by plants, and its application is important for improving crop yield and quality (Blumenthal et al., 2008). Zhi-liang and Chang-yan (2011) found that insufficient water or excessive nitrogen supply limited the accumulation of dry matter in crops, leading to early senescence and lower yields. Jalpa et al. (2020) demonstrated that excessive nitrogen application did not significantly increase the yields of greenhouse vegetables, whereas Li et al. (2015) found that appropriate water and nitrogen management is important to improve the efficiency of water and nitrogen use, optimize inputs, and fully exploit the synergistic effects of water and nitrogen. Sanford et al. (2021) found that reducing nitrogen application inhibits the growth and development of sunflowers, affecting yield formation and reducing irrigation water use efficiency. In contrast, appropriate water and nitrogen ratios can stabilize yield while decreasing irrigation and nitrogen inputs, thereby improving the water use efficiency of sunflowers. Quinoa, as a cold-tolerant, drought-tolerant, and barren-tolerant high-nutrient crop, holds significant potential for cultivation in the arid regions of Northwest China. At present, domestic and foreign research on water–nitrogen intercropping mainly focuses on grain crops such as maize and rice (Liu et al., 2019; Wang et al., 2023; Jin et al., 2024), with fewer studies examining the threshold effects of water–nitrogen coupling in quinoa and its impact on quality regulation. In particular, systematic research on the specific threshold effects of water–nitrogen interactions in quinoa cultivation under arid conditions, as well as the underlying regulatory mechanisms influencing grain quality, remains limited. Compared with conventional cereal crops, quinoa exhibits distinct physiological traits for drought resistance and superior nutrient use efficiency, indicating that its water and nitrogen requirements, as well as their interactive effects, may differ significantly. Based on these observations, this study hypothesizes that optimal water and nitrogen (*W*–*N*) combinations can simultaneously maximize yield, maintain grain quality, and enhance water and nitrogen use efficiency through tailored supply strategies. To test this hypothesis, a two-factor field experiment was conducted in conjunction with response surface methodology to elucidate the synergistic effects of water and nitrogen on quinoa yield, quality, and resource use efficiency. The findings are expected to provide a scientific basis and technical guidance for sustainable and green quinoa production in arid regions.

## 2 Materials and methods

### 2.1 Profile of the test area

**Figure 1:**
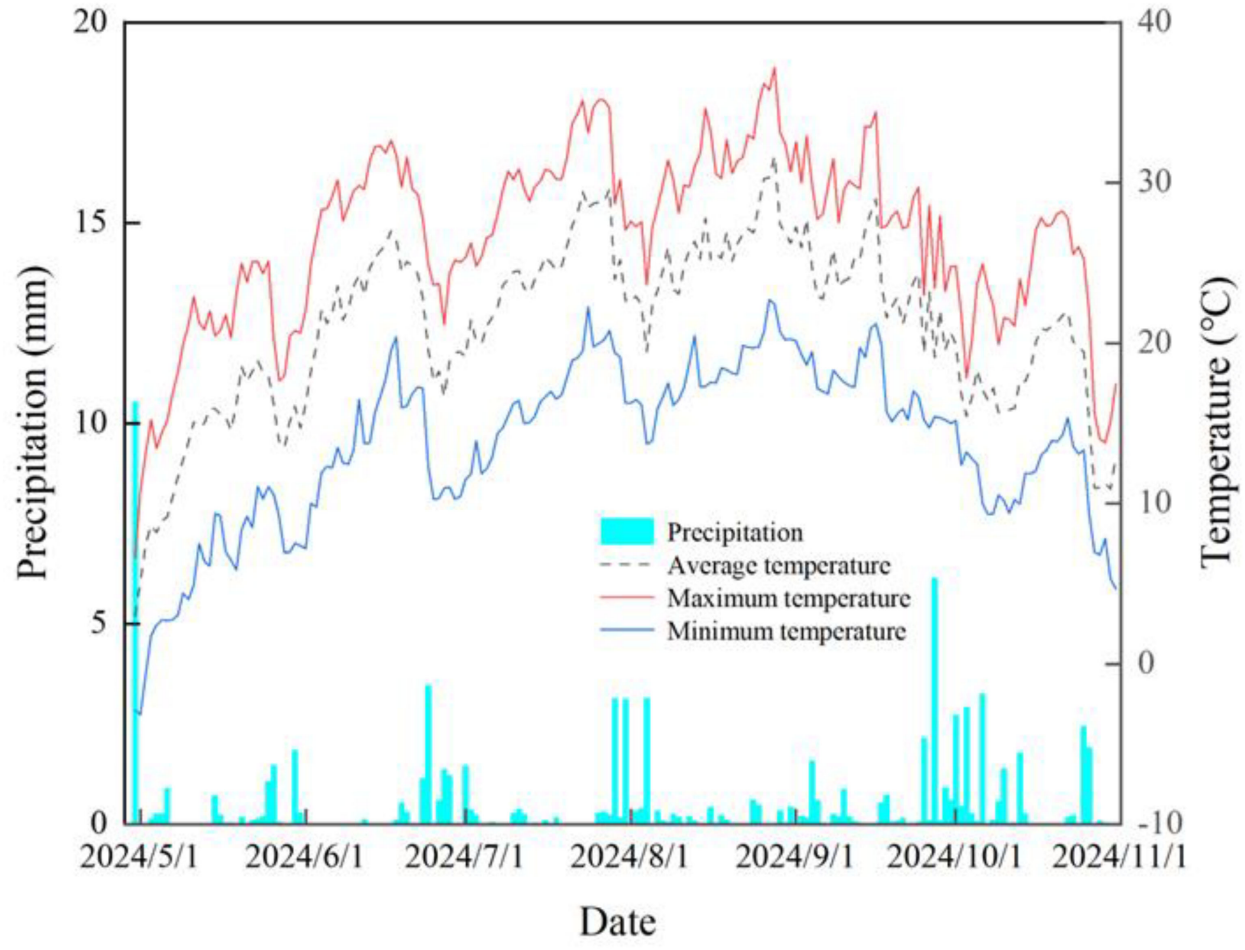
Rainfall and temperature in Baicheng County, Aksu Prefecture, in 2024.

The experiment was carried out from April to September 2024 at the National Brown Desert Soil Experimental Station (41°47′N, 81°54′E) in Baicheng County, Aksu Region, Xinjiang. The site has a temperate continental arid climate, with an average annual temperature of 7.8°C, a frost-free period of 133–163 days, an annual average of 2,780.1 h of sunshine, and a mean annual precipitation of 94 mm (**Figure 1**). The soil texture in the experimental area is clayey loam, and the basic physicochemical properties of the tillage-layer soil prior to the experiment are shown in **Table 1**.

**Table 1:** Physical and chemical properties of the tested soil.

Soil layer (cm)	Organic matter (g/kg)	Total N (g/kg)	Total P (g/kg)	Available N (mg/kg)	Available P (mg/kg)	Available K (mg/kg)	pH	(m/cm^3^)
0–20	16.90	1.68	1.53	17.02	14.7	409.06	8.22	1.44
20–40	10.65	1.43	1.21	14.33	13.27	347.33	8.14	1.44

### 2.2 Test design

The test variety was Jiaqi 510, provided by Shanxi Jiaqi Agricultural Science and Technology Co. The fertilizers used were urea (*N*: 46%), calcium superphosphate (P_2_O_5_: 16%), and potassium sulfate (K_2_O: 50%). A drip irrigation system was used, consisting of a water meter, fertilizer tank, PE hose, patch-type drip irrigation tape, with a drip head spacing of 25 cm. The ground film was polyethylene, 0.8 m in width and 0.008 mm in thickness. Water and fertilizer applications were controlled through the fertilizer tank and water meter throughout the experimental period. The depth of each microarea was 100 cm, with 90 cm filled with soil. Each microarea contained 2,600 kg of dry soil, divided into two layers: the lower layer is filled with subsoil from 30 to 90 cm of the field, and the upper layer was composed of tillage soil from 0 to 20 cm of the field. The bottom of each microarea was in direct contact with the natural soil.

**Figure 2:**
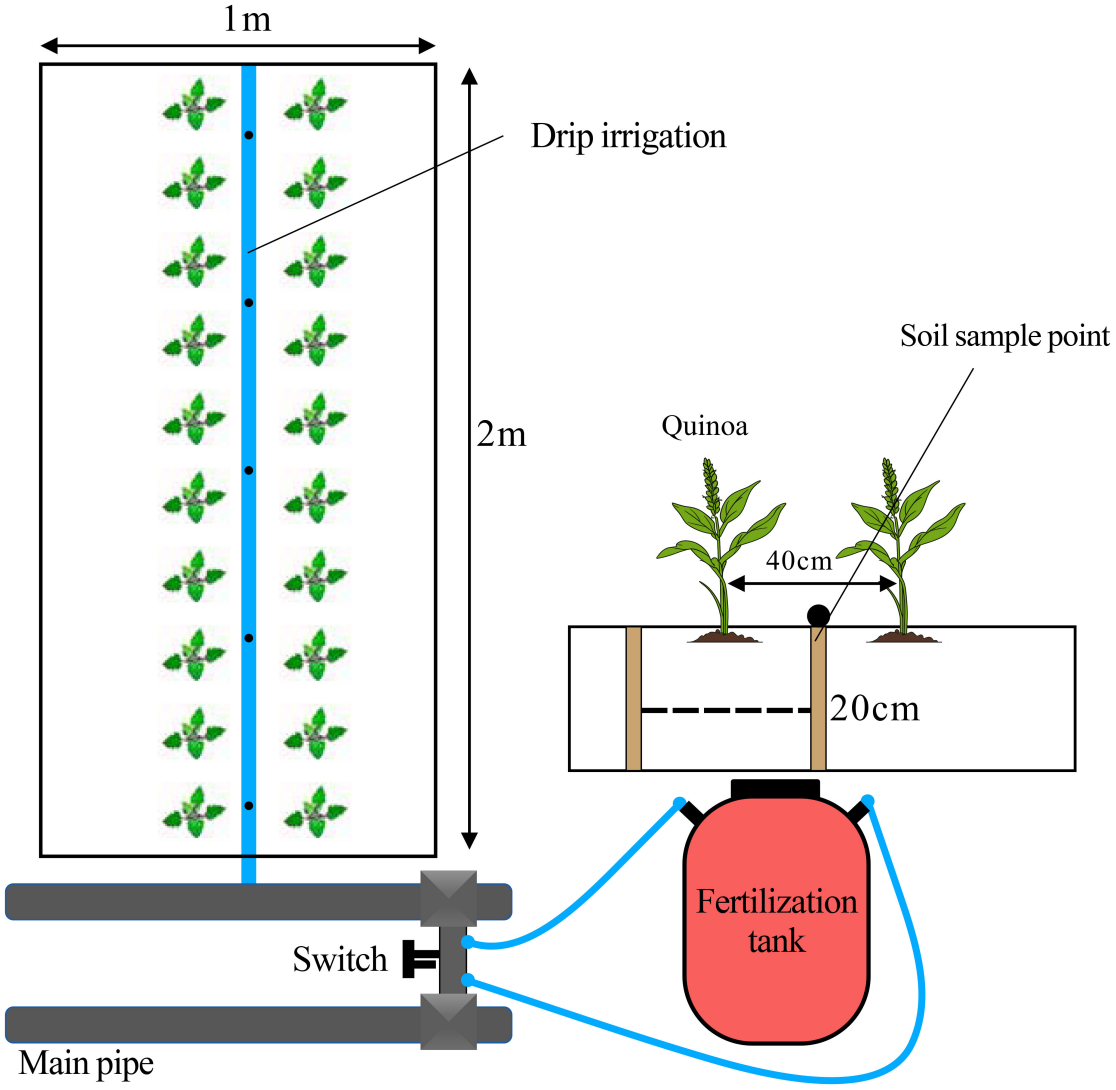
Microarea quinoa cultivation model.

The experiment was set up with 15 treatments, three replications, and a total of 45 plots, with each plot measuring 1 m × 2 m = 2 m^2^. The experiment was designed as a two-factor interaction study, with irrigation and fertilization treatments applied according to the methodology of Bahrami et al. (2022) and adapted to reflect local farmers’ irrigation and fertilization practices. The two factors were irrigation quota (*W*) and nitrogen application (*N*). Three irrigation levels were used: 2,100, 2,700, and 3,300 m^3^/ha, noted as *W*1, *W*2, and *W*3; and five nitrogen (*N*) application levels were used: 0, 100, 125, 150, 175 kg/ha, recorded as *N*0, *N*1, *N*2, *N*3, and *N*4. Quinoa was sown with a row spacing of 40 cm, plant spacing of 20 cm, sowing depth of 1 cm, using hole sowing with five seeds per hole, resulting in a planting density of 100,050 plants/ha. The seeds were sown on 22 April 2024. Irrigation and fertilization were carried out at the seedling stage (20 May 2024), spike stage (25 June 2024), and irrigated and fertilized at the filling stage (15 August 2024). Nitrogen fertilizer was applied 30% as a basal dose, with the remaining 70% applied in three splits with irrigation (10% at the seedling stage, 30% at the spike stage, and 30% at the grouting stage). Phosphorus and potash fertilizers were applied 20% as basal fertilizer, with the remaining 80% applied with irrigation at the spike and grouting stages. When the seedling reached 6–9 cm after interseeding, three strong seedlings were selected per column. When seedlings grew to 10–15 cm, one healthy seedling per hole without pests or diseases was retained. The planting pattern is shown in **Figure 2**.

### 2.3 Indicators and methods of project determination

Plant samples were collected at the seedling stage (25 May), seed-bearing panicle stage (10 July), grouting stage (20 August), and maturity stage (16 September). Quinoa plants were washed with deionized water, dried with absorbent paper, and separated into above- and belowground parts. The samples were placed in kraft paper bags, killed at 105°C for 30 min, and then dried at 75°C for 48 h to constant weight. The biomass of quinoa was subsequently determined (Jin et al., 2024).

Sampling was carried out when more than 80% of the quinoa leaves had turned yellow, some had started to fall, and the seeds had hardened. Ten plants per treatment were randomly sampled at uniform growth for manual harvesting. After drying, each sample was threshed to determine individual grain weight, thousand-kernel weight, and yield. Yield per treatment was calculated by threshing and sun-drying the harvested quinoa. Additionally, five plants per treatment were randomly selected at maturity, their measurements averaged, and the results converted to yield per hectare.

Starch content was determined using the spinning method (National Standard GB 5006-85); fat content was measured by the Soxhlet extraction method; protein content was determined using the Kjeldahl method.

### 2.4 Calculation formula of each index

1. Water use efficiency (WUE, kg/m^3^) denotes the efficiency with which water is utilized in the formation of seed yield and is calculated according to Equations (1–3).

(1) $$\text{WUE}=\frac{\text{Y}}{\text{ET}}$$

(2) ET=P+W+S

(3)  ΔS=pre-sowing soil water storage－post-harvest soil water storage

Where *Y* is the quinoa grain yield (kg/ha), ET is water consumption (m^3^/ha), *P* is rainfall (mm), *W* is irrigation water (m^3^), and Δ*S* is the change in soil water storage between presowing and postharvest (mm).

2. Nitrogen agronomic efficiency and nitrogen partial factor productivity.

(4) $$\text{NAE}=\frac{\text{yield in N application area }-\text{yield in no N application area}}{\text{Nitrogen application}}$$

(5) $$\;\text{NPFP}=\frac{\text{yield in nitrogen application area}}{\text{amount of nitrogen applied}}$$

Where NAE is the nitrogen agronomic efficiency (kg/kg) and NPFP is nitrogen partial factor productivity (kg/kg). Here, NAE is calculated by Equation (4) and NPFP is calculated by Equation (5).

### 2.5 Data processing

Preliminary data organization was performed using Microsoft Excel 2018. Statistical analyses, including two-way analysis of variance (ANOVA), correlation analysis, and significance testing, were conducted using SPSS 27.0. All graphs were generated using Origin 2021.

## 3 Results

### 3.1 Effect on dry matter quality of quinoa under different irrigation and nitrogen application treatments

The analysis of variance results (**Table 2**) revealed that both irrigation level and nitrogen application rate had highly exerted highly significant effects on dry matter accumulation in quinoa across all growth stages (*p* < 0.01). Distinct patterns of dry matter accumulation were observed under different water–nitrogen combinations. Under identical irrigation conditions, dry matter accumulation initially increased and then declined with increasing nitrogen application. The highest dry matter accumulation was recorded under the *W*2*N*3 treatment (moderate irrigation with high nitrogen in reclaimed water), which was significantly greater than that under other treatment combinations (*p* < 0.05). This suggests that optimal dry matter yield is achieved through a combination of moderate water supply and sufficient nitrogen nutrition. In the high evapotranspiration environment of southern Xinjiang, irrigation level serves as the primary determinant of yield formation, followed by the interactive effect of water and nitrogen. The dry matter mass under low irrigation (*W*1) and zero nitrogen (*N*0) was lower than that under medium (*W*2) and high (*W*3) irrigation levels, indicating that increased irrigation improves nitrogen use efficiency and enhances dry matter accumulation. Under *W*1 conditions, dry matter mass increased progressively with nitrogen application, implying that supplemental nitrogen can partially compensate for biomass limitations under water-deficient conditions. However, under medium and high irrigation levels (*W*2 and *W*3), exceeding a nitrogen application rate of 150 kg/ha led to a reduction in dry matter accumulation by 12.6%–18.9%, demonstrating a clear threshold effect in nitrogen uptake; excessive nitrogen application thus suppresses dry matter accumulation in quinoa.

**Table 2:** The influence of different irrigation nitrogen application treatments on the dry matter quality of quinoa.

Irrigation	Nitrogen	Seedling stage (g)	Seed-bearing panicles stage (g)	Grouting stage (g)	Maturity stage (g)
*W*1	*N*0	0.13 ± 0.01 d	32.88 ± 2.58 h	56.23 ± 3.84 g	101.2 ± 1.49 l
	*N*1	0.25 ± 0.02 d	42.65 ± 2.61 f	99.83 ± 0.63 e	112.46 ± 3.77 g
	*N*2	0.43 ± 0.18 c	44.11 ± 3.89 ef	101.02 ± 2.32 e	135.24 ± 2.86 e
	*N*3	0.45 ± 0.30 c	46.53 ± 1.66 de	113.31 ± 2.84 c	137.49 ± 2.17 de
	*N*4	0.45 ± 0.06 c	47.65 ± 2.17 cd	117.26 ± 2.85 bc	141.93 ± 1.63 d
*W*2	*N*0	0.15 ± 0.05 d	38.41 ± 1.17 g	59.3 ± 3.35 g	106.62 ± 6.59 l
	*N*1	0.26 ± 0.03 d	46.64 ± 1.53 de	101.2 ± 2.19 e	121.11 ± 1.28 f
	*N*2	0.77 ± 0.04 ab	48.56 ± 0.85 bcd	106.49 ± 0.89 d	156.85 ± 3.91 b
	*N*3	0.80 ± 0.01 a	51.24 ± 0.92 ad	124.42 ± 3.02 a	164.17 ± 3.92 a
	*N*4	0.74 ± 0.14 ab	49.92 ± 1.18 abc	127.26 ± 2.16 a	158.17 ± 3.62 b
*W*3	*N*0	0.19 ± 0.05 d	37.68 ± 2.52 g	60.71 ± 1.43 f	111.96 ± 3.23 gh
	*N*1	0.26 ± 0.06 d	48.26 ± 0.13 cd	105.94 ± 4.15 d	126.6 ± 4.17 f
	*N*2	0.77 ± 0.01 ab	51.61 ± 0.64 a	117.26 ± 2.85 bc	157.62 ± 4.51 b
	*N*3	0.77 ± 0 ab	51.33 ± 1.02 ab	118.17 ± 1.91 b	164.77 ± 5.01 a
	*N*4	0.63 ± 0.11 b	50.32 ± 0.69 abc	118.53 ± 5.39 b	148.41 ± 3.97 c
*F*(*W*)		^**^	^**^	^**^	^**^
*F*(*N*)		^**^	^**^	^**^	^**^
*F*(*W* × *N*)		^*^	ns	^**^	^**^

^*^p < 0.05; ^**^p < 0.01. Data are means ± SD (*n* = 3). ns, non-significant.

Different lowercase letters indicate significant differences at the 0.05 probability level (P<0.05), determined by one-way analysis of variance (ANOVA) and Duncan's post hoc test for significance.

### 3.2 Effects of different irrigated nitrogen treatments on quinoa yield and components of yield

Thousand-kernel weight and individual grain weight are core elements of yield composition. ANOVA results (**Table 3**) showed that the range of thousand-kernel weight among treatments was 2.22–3.67 g, while individual grain weight ranged from 3.03 to 44.94 g, with differences among treatments being highly significant (*p* < 0.01). Under the same nitrogen application condition, the thousand-kernel weight and individual grain weight of *W*2 and *W*3 irrigation treatments were significantly higher than those of the *W*1 treatment, indicating that increased irrigation can significantly enhance both the thousand-kernel weight and individual grain weight of quinoa.

**Table 3:** Effects of different irrigation nitrogen treatments on quinoa yield and its constituent factors.

Irrigation	Nitrogen	Thousand-grain weight (g)	Individual grain weight (g)	Yield (kg/ha)
*W*1	*N*0	2.22 ± 0.01 d	3.03 ± 0.14 h	272.75 ± 12.2 h
	*N*1	2.27 ± 0.05 d	9.9 ± 0.33 g	890.49 ± 29.4 g
	*N*2	2.63 ± 0.04 c	33.71 ± 1.07 e	3,000.51 ± 144.44 e
	*N*3	3.15 ± 0.1 b	33.93 ± 1.78 e	3n300.3 ± 88.74 d
	*N*4	3.16 ± 0.02 b	34.78 ± 0.26 e	3n467.11 ± 109.31 c
*W*2	*N*0	2.23 ± 0.01 d	3.27 ± 0.35 h	293.71 ± 31.06 h
	*N*1	2.61 ± 0.05 c	23.33 ± 0.58 f	2,097.2 ± 51.85 f
	*N*2	3.21 ± 0.01 b	44.94 ± 0.39 a	4,040.65 ± 34.81 a
	*N*3	3.67 ± 0.57 a	44.43 ± 1.78 a	4,044.42 ± 84.44 a
	*N*4	3.15 ± 0.1 b	41.26 ± 3.24 c	3,709.56 ± 290.97 b
*W*3	*N*0	2.25 ± 0.07 d	3.46 ± 0.24 h	311.02 ± 21.44 h
	*N*1	2.67 ± 0.03 c	23.82 ± 0.9 f	2,141.44 ± 80.8 f
	*N*2	3.21 ± 0.01 b	42.61 ± 0.26 bc	4,045.95 ± 59.83 a
	*N*3	3.23 ± 0 b	43.2 ± 0.18 a	4,024.32 ± 35.42 a
	*N*4	3.09 ± 0.04 b	38.66 ± 1.13 d	3,640.78 ± 49.13 b
*F*(*W*)		^*^	^**^	^**^
*F*(*N*)		^**^	^*^	^**^
*F*(*W*×*N*)		^**^	^**^	^**^

^*^p < 0.05; ^**^p < 0.01. Data are means ± SD (*n* = 3).

Different lowercase letters indicate significant differences at the 0.05 probability level (P<0.05), determined by one-way analysis of variance (ANOVA) and Duncan's post hoc test for significance.

Crop yield is the main index for evaluating the advantages and disadvantages of water–nitrogen coupling (Ma et al., 2024). Under *W*1 irrigation conditions, the yield gradually increased with increasing nitrogen application, indicating that an increase in nitrogen application under insufficient irrigation could improve the yield of quinoa. The yield of quinoa under medium and high irrigation levels (*W*2, *W*3) showed an increasing and then decreasing trend with increasing *N* application, indicating that when *N* application exceeded a certain range (150 kg/ha), further *N* fertilization would inhibit yield improvement.

### 3.3 Analysis of the effect of water–nitrogen interaction on yield

**Figure 3:**
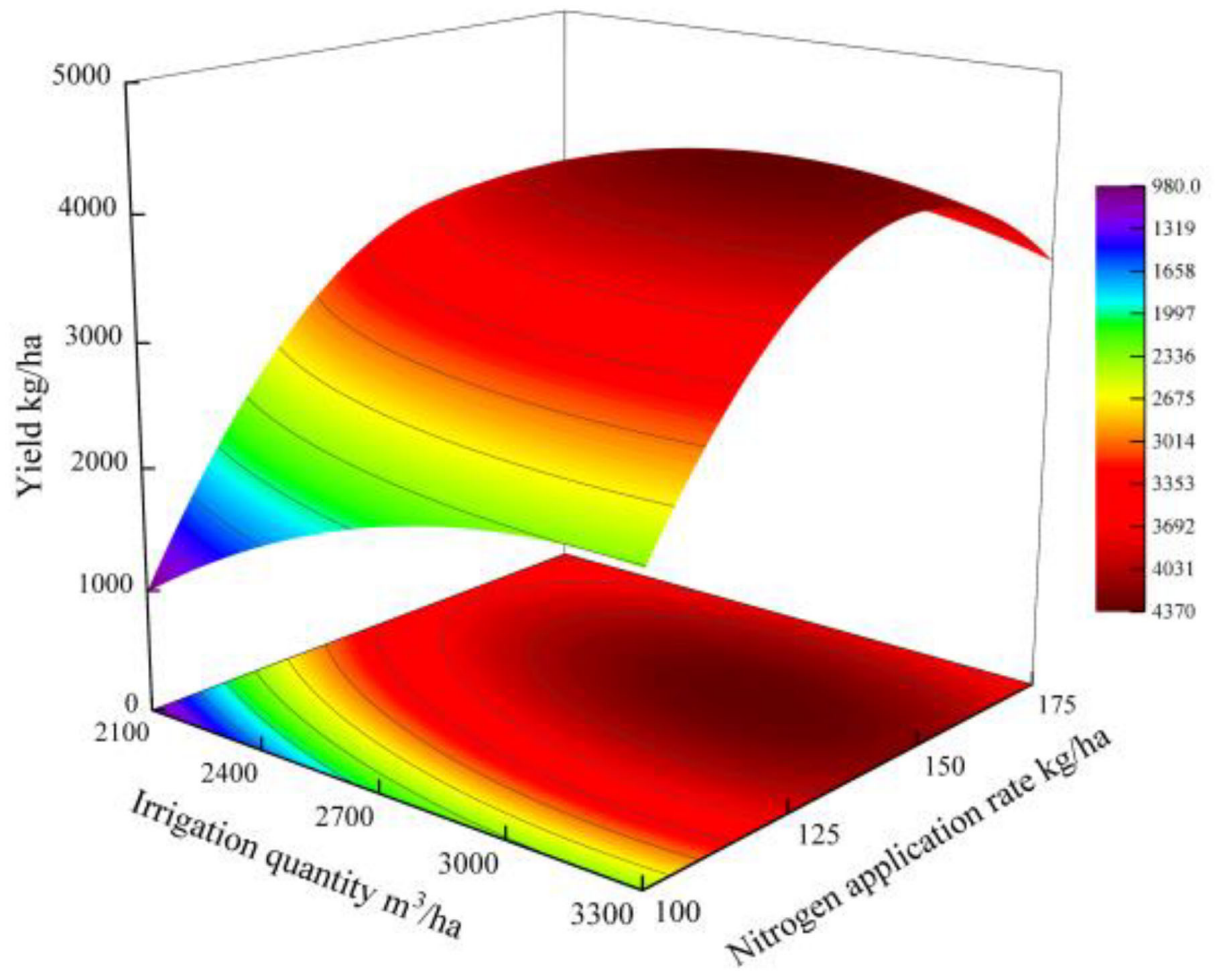
Analysis of the impact of water–nitrogen interaction on yield.

Regression model of quinoa yield (*Y*) under varying irrigation (*W*) and *N*) regimes: *Y* = 988.30 + 2,967.89*W* + 7,149.87*N* – 1,636.38*W*^2^ – 1,065.98*WN* – 4,882.19*N*^2^ (*R*^2^ = 0.945, *p* < 0.01). The theoretical maximum yield (4,363.91 kg/ha) occurred at *W* = 2,931.59 m^3^/ha and *N* = 149.24 kg/ha. The quadratic term coefficients *W*^2^ (− 1,636.38) and *N*^2^ (− 4,882.19) were both negative, confirming that the yield response surface was characterized by a typical downward-opening paraboloid (**Figure 3**). The interaction term, *WN* coefficient (− 1,065.98), indicated that the water–nitrogen synergistic effect had a threshold response characteristic. The highest yield of 4,363.91 kg/ha was predicted when the irrigation rate was 2,931.59 m^3^/ha and the *N* application rate was 149.24 kg/ha. The regression model (*R*^2^ = 0.945) constructed in this study revealed the nonlinear regulation mechanism of the water–nitrogen interaction on quinoa yield. It is recommended to maintain irrigation at 2,800–3,100 m^3^/ha and adjust nitrogen application to 140–160 kg/ha in production practice to achieve higher yields.

### 3.4 Effects of different irrigation nitrogen application treatments on quinoa quality

The results of ANOVA on the effect of water–nitrogen interactions on quinoa quality showed that the effects of irrigation volume (**Table 4**), nitrogen application, and their interactions reached highly significant (*p* < 0.01) or significant levels (*p* < 0.05) on the protein and starch contents of the kernel, whereas the effect of irrigation treatments alone on the fat content was not significant (*p* > 0.05). There were significant differences (*p* < 0.05) in the response patterns of nutrients under different water gradients. Under *W*1 (low irrigation) conditions, the fat, protein, and starch contents of the *N*4 treatment were significantly increased by 64.28%, 18.88%, and 29.87%, respectively, compared with those of the *N*0 treatment (*p* < 0.01), showing a tendency to increase with the rise in nitrogen application. Under *W*2 (medium irrigation) and *W*3 (high irrigation) conditions, the contents of all three nutrients showed significant single-peak curves with the increase in nitrogen application, in which the *N*2 and *N*3 treatments showed optimal effects (*p* < 0.05), with the contents of fat, protein, and starch increased by 85.04%–97.67%, 22.20%–23.94%, and 57.46%–58.13%, respectively, compared with the treatment with no nitrogen (*N*0).

**Table 4:** Effects of different irrigation nitrogen application treatments on quinoa quality.

Irrigation	Nitrogen	Fat (g/kg)	Protein (g/kg)	Starch (g/kg)
*W*1	*N*0	28.53 ± 8.11 e	156.94 ± 4.69 i	369.78 ± 8.69 i
	*N*1	33.99 ± 10.44 de	164.35 ± 1.25 h	403.97 ± 2.83 h
	*N*2	45.19 ± 2.54 bc	181.1 ± 2.63 e	429.01 ± 9.69 f
	*N*3	45.61 ± 1.9 bc	186.57 ± 1.07 cd	452.43 ± 13.07 e
	*N*4	46.87 ± 4.04 bc	184.71 ± 0.75 de	480.23 ± 6.15 d
*W*2	*N*0	27.86 ± 0.81 e	160.32 ± 3.04 hi	369.06 ± 5.4 i
	*N*1	39.71 ± 5.19 cd	175.3 ± 2.04 f	414.99 ± 3.17 g
	*N*2	55.06 ± 0.4 a	198.69 ± 4.47 a	582.55 ± 5.31 a
	*N*3	55.9 ± 1.96 a	197.43 ± 0.85 ab	583.61 ± 3.1 a
	*N*4	50.63 ± 7.28 ab	193.94 ± 0.98 b	569.66 ± 6.82 b
*W*3	*N*0	29.67 ± 4.61 e	162.88 ± 4.35 h	372.13 ± 3.63 i
	*N*1	32.33 ± 3.65 de	170.03 ± 3.66 g	420.8 ± 5.73 fg
	*N*2	51.47 ± 5.12 ab	189.68 ± 1.3 c	581.23 ± 7.29 a
	*N*3	54.9 ± 2.24 a	199.05 ± 3.71 a	585.96 ± 2.45 a
	*N*4	44.21 ± 1.52 bc	181.5 ± 1.9 e	547.63 ± 10.86 c
*F*(*W*)		ns	^*^	^**^
*F*(*N*)		^**^	^**^	^**^
*F*(*W*×*N*)		^**^	^**^	^**^

^*^p < 0.05; ^**^p < 0.01. Data are means ± SD (*n* = 3). ns, non-significant.

Different lowercase letters indicate significant differences at the 0.05 probability level (P<0.05), determined by one-way analysis of variance (ANOVA) and Duncan's post hoc test for significance.

### 3.5 Effects of different irrigation and nitrogen application treatments on water and nitrogen use efficiency in quinoa

The results of ANOVA showed (**Table 5**) that irrigation level, nitrogen application, and their interaction had highly significant effects on WUE, NAE and NPFP (*p* < 0.01). Under the same irrigation level, with the increase in nitrogen fertilizer dosage, water use efficiency as a whole showed a trend of first increasing and then decreasing, with the *W*2*N*3 treatment having the highest water use efficiency, followed by the *W*2*N*2 treatment. Nitrogen agronomic utilization reflects the yield-increasing effect of nitrogen dosage, and nitrogen fertilizer bias productivity reflects the combined effect of local soil base nutrient level and fertilizer application (Ma et al., 2024). NAE and NPFP exhibited the same pattern of change, both showing a trend of first increasing and then decreasing with the rise in nitrogen agronomic application. NAE and NPFP showed the highest performance under *W*2*N*2 treatment, indicating that nitrogen made the greatest contribution to the increase in quinoa yield. With the increase in nitrogen fertilizer application, nitrogen agronomic efficiency, nitrogen physiological utilization, and nitrogen harvest index showed a decreasing trend, indicating that nitrogen contributed to the increase of seed grain yield. The contribution of nitrogen to the increase in seed yield was significantly reduced; with the increase in nitrogen application, the NPFP of quinoa showed a decreasing trend, indicating that the contribution of nitrogen application to the yield of quinoa showed a decreasing trend.

**Table 5:** Effects of different irrigation nitrogen application treatments on water–nitrogen utilization efficiency.

Irrigation	Nitrogen	WUE (g/kg)	NAE (kg/kg)	NPFP (kg/kg)
*W*1	*N*0	0.13 ± 0.01 j	–	–
	*N*1	0.42 ± 0.01 i	6.18 ± 0.21 h	8.9 ± 0.29 g
	*N*2	1.28 ± 0.01 d	19.34 ± 0.17 d	21.52 ± 0.14 d
	*N*3	1.47 ± 0.03 b	18.78 ± 0.46 efg	20.6 ± 0.46 f
	*N*4	1.51 ± 0.03 b	16.54 ± 0.33 g	18.1 ± 0.33
*W*2	*N*0	0.11 ± 0.01j	–	–
	*N*1	0.78 ± 0.02 g	18.03 ± 0.79 f	20.97 ± 0.52 de
	*N*2	1.48 ± 0.02 b	29.7 ± 0.67 a	32.05 ± 0.49 a
	*N*3	1.59 ± 0.01 a	26.7 ± 0.23 b	28.66 ± 0.16 b
	*N*4	1.37 ± 0.11 c	19.52 ± 1.58 d	21.2 ± 1.66 de
*W*3	*N*0	0.09 ± 0.01 j	–	–
	*N*1	0.65 ± 0.02 h	18.3 ± 0.94 ef	21.41 ± 0.81 de
	*N*2	1.23 ± 0.02 e	29.88 ± 0.31 a	32.37 ± 0.48 a
	*N*3	1.23 ± 0.01 e	24.76 ± 0.11 c	26.83 ± 0.24 c
	*N*4	1.1 ± 0.01 f	19.03 ± 0.3 de	20.8 ± 0.28 de
*F*(*W*)		^**^	^**^	^**^
*F*(*N*)		^**^	^**^	^**^
*F*(*W*×*N*)		^**^	^**^	^**^

^*^p < 0.05; ^**^p < 0.01. Data are means ± SD (*n* = 3).

Different lowercase letters indicate significant differences at the 0.05 probability level (P<0.05), determined by one-way analysis of variance (ANOVA) and Duncan's post hoc test for significance.

### 3.6 Analysis of the impact of water–nitrogen interaction on water use efficiency

In order to investigate the dynamic and continuous change law of WUE, NAE, and NPFP of quinoa with the change of irrigation level and nitrogen fertilizer application, regression analyses were carried out by taking water use efficiency, nitrogen fertilizer agronomic utilization, and nitrogen fertilizer bias productivity as the dependent variables, and irrigation amount and nitrogen application amount as the independent variables, respectively. A response surface model of WUE, NAE, and NPFP was constructed, and its extreme effects, as well as their agronomic thresholds, were analyzed.

**Figure 4:**
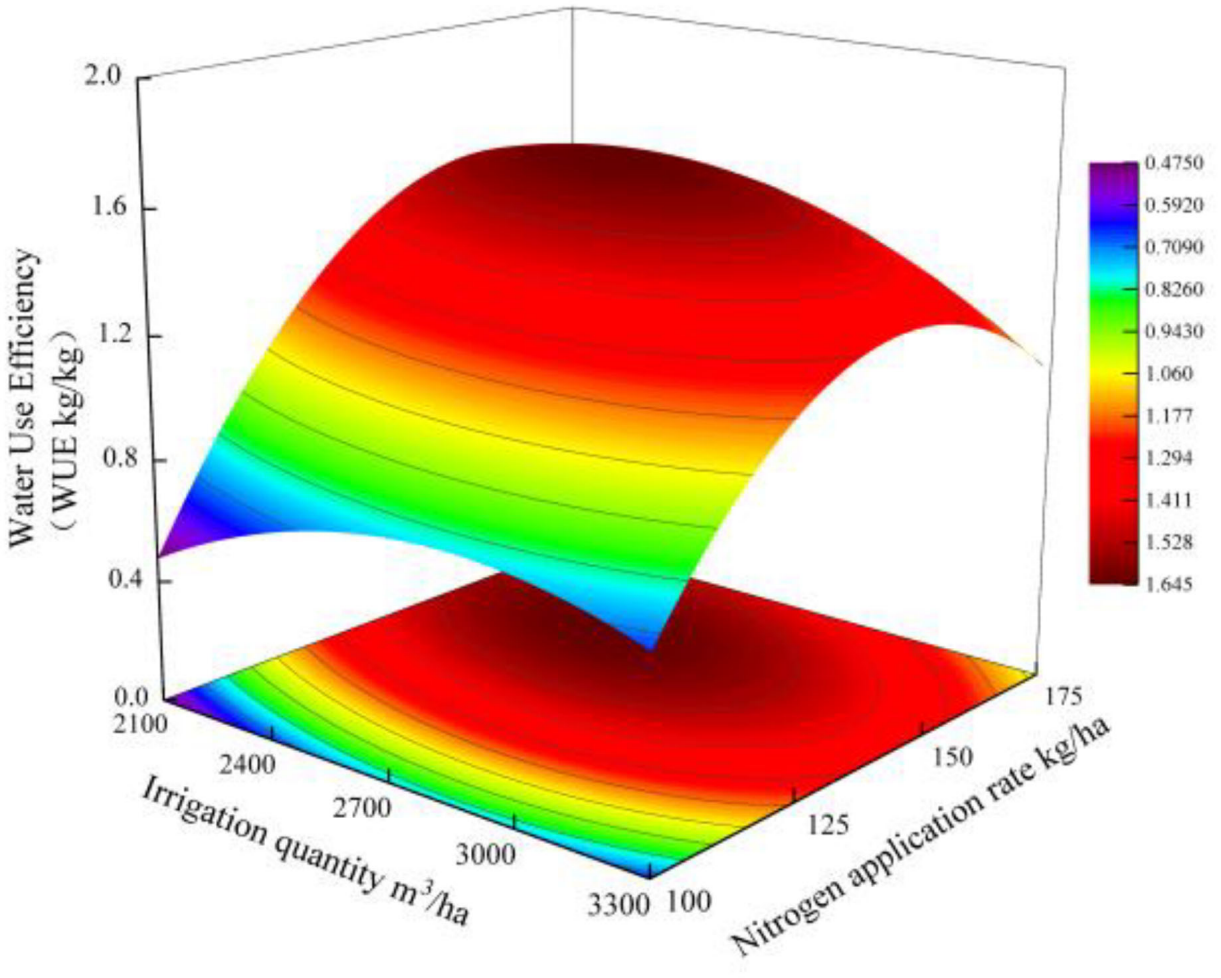
Analysis of the impact of water–nitrogen interaction on water use efficiency.

WUE = 0.48 + 0.98*W* + 2.81*N* − 0.79W^2^ − 0.63*WN* − 1.83N^2^ (*R*^2^ = 0.965). The mathematical maximum value of 1.64 corresponds to a nitrogen application of 153.31kg/ha and an irrigation volume of 2,506.15 m^3^/ha. The quadratic coefficients *W*^2^ (− 0.79) and *N*^2^ (− 1.83) were both negative, confirming that the WUE response surface was a typical downward-opening paraboloid. By solving the system of partial derivative equations, the global maximum points were determined: *W* = 2,506.15 m^3^/ha and *N* = 153.31 kg/ha, corresponding to a theoretical maximum WUE of 1.64 g/kg. Marginal effect analysis showed that the rate of decrease in WUE was 0.35% for irrigation above 3000 m^3^/ha, and 0.52% for N application above 180 kg/ha (**Figure 4**).

### 3.7 Analysis of the impact of water–nitrogen interaction on nitrogen use efficiency

**Figure 5:**
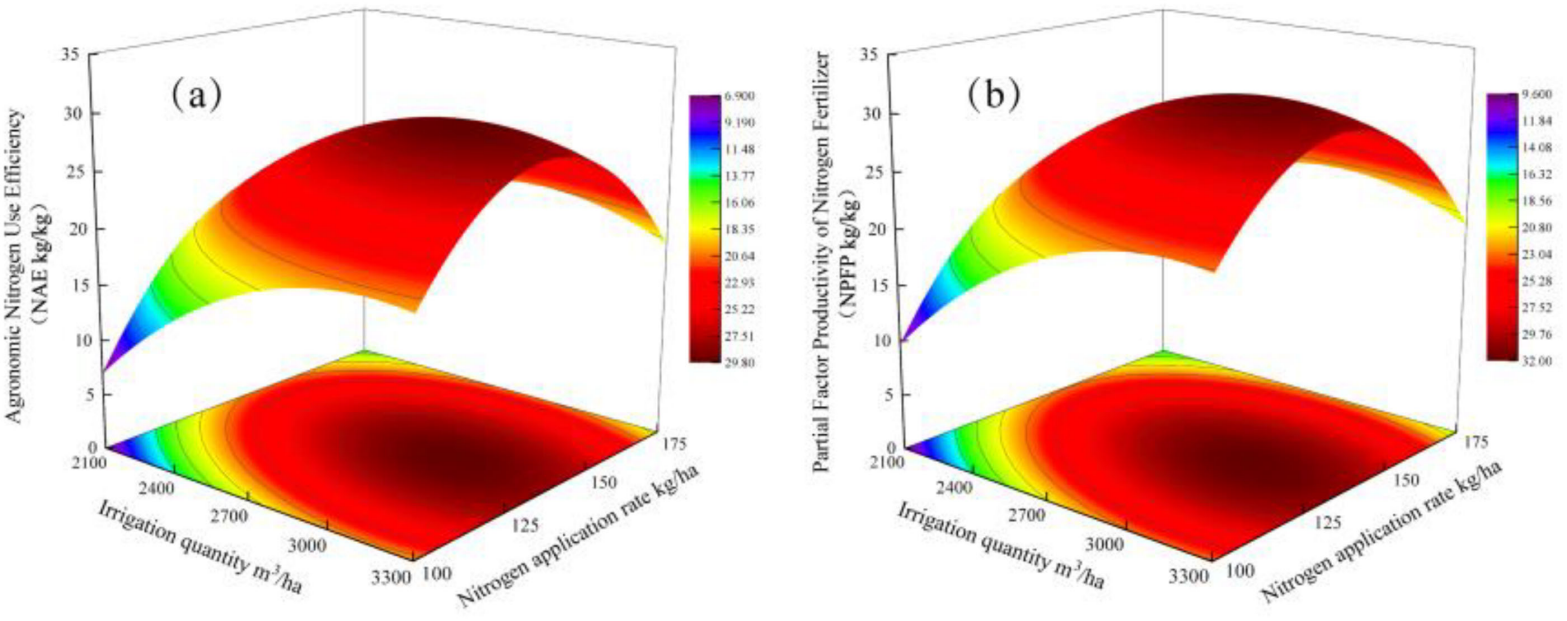
**(a)** Analysis of the impact of water–nitrogen interaction on the nitrogen agronomic efficiency. **(b)** Analysis of the impact of water–nitrogen interaction on the nitrogen partial factor productivity.

NAE = 6.95 + 30.35*W* + 46.59*N* − 17.55*W*^2^− 10.04*WN* − 38.67*N*^2^ (*R*^2^ = 0.925). The quadratic term coefficients *W*2 (− 17.55) and *N*2 (− 38.67) are negative, indicating that the NAE response surface is an open downward-opening paraboloid, with a global maximum. NAE reached the peak value of 29.72 kg/kg when *W* = 2,962.89 m^3^/ha and *N* = 138.18 kg/ha, the NAE reached a peak value of 29.72 kg/kg. The interaction term *WN* coefficient (− 10.04) revealed the nonlinear characteristics of the water–nitrogen synergistic effect, indicating the existence of a threshold response of water–nitrogen ratios (**Figure 5a**). NPFP = 9.68 + 30.78*W* + 44.74*N* − 17.61*W*^2^ − 10.21*WN* − 37.98*N*^2^ (*R*^2^ = 0.925). The model extremes were located at *W* = 2,978.33 m^3^/ha and *N* = 136.80 kg/ha, corresponding to a maximum NPFP value of 31.92 kg/kg (**Figure 5b**). The regression model showed that the water–nitrogen interaction had an effect on (negative coefficients of *W* and *N* terms) *N* fertilizer agronomic utilization and *N* fertilizer bias productivity, as reflected by the negative coefficients of the *W* and *N* terms. Exceeding the thresholds (*W* > 3,000 m^3^/ha or *N* > 150 kg/ha) led to reductions in both *N* fertilizer agronomic utilization and *N* fertilizer bias productivity. Therefore, it is necessary to dynamically adjust the water and nitrogen inputs based on local precipitation and nitrogen monitoring to simultaneously optimize the yield and nitrogen use efficiency.

### 3.8 Correlation analysis of water and nitrogen interaction on indicators

**Figure 6:**
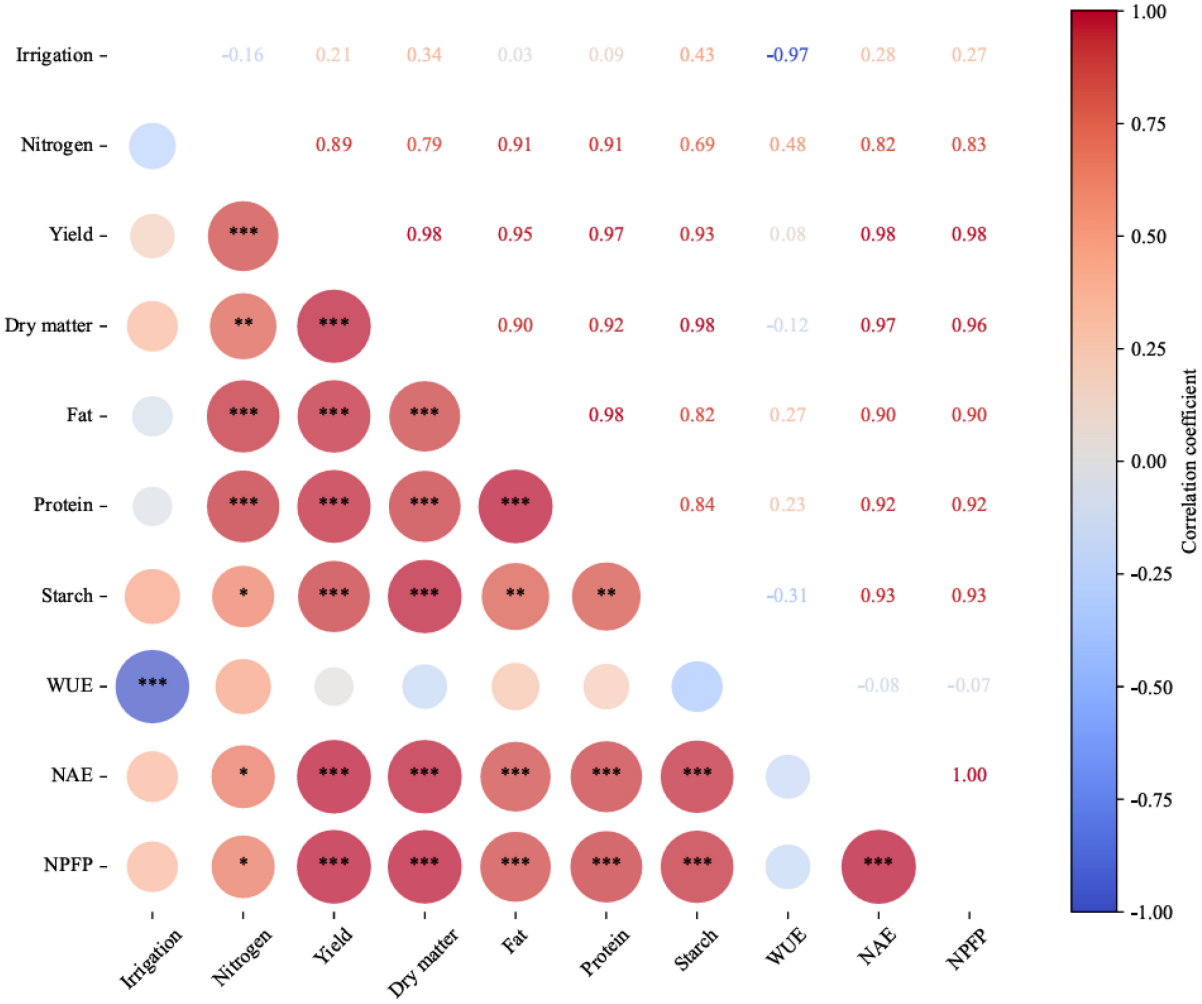
Correlation analysis of each index. ^*^p < 0.05; ^**^p < 0.01; ^***^p < 0.001. Blue indicates a negative correlation between the two indicators, red indicates a positive correlation, and the depth of the color represents the magnitude of the correlation coefficient.

The results of the correlation analysis (**Figure 6**) showed that irrigation level (*W*) was highly significantly negatively correlated (*p* < 0.01) with quinoa WUE but had no significant effect on the other indicators. Nitrogen application (*N*) was highly significantly negatively correlated (*p* < 0.01) with yield, dry matter, fat, and protein content, and significantly negatively correlated (*p* < 0.05) with starch, NAE, and NPFP, suggesting that excessive nitrogen fertilizer application substantially inhibited quinoa yield movement and nitrogen use efficiency. Neither factor had a significant reciprocal effect on WUE. Experimental data showed that high irrigation levels reduced quinoa water use efficiency, whereas excessive nitrogen fertilization inhibited yield improvement and negatively affected quinoa quality. Optimizing the water and nitrogen ratio is therefore essential in quinoa production to balance yield formation and resource use efficiency, while avoiding excessive nitrogen application.

## 4 Discussion

As a drought-tolerant crop, quinoa is particularly sensitive to nitrogen, making integrated water–nitrogen regulation especially important in its production (Dehghanian et al., 2024). Gao et al. (2024) showed that reasonable regulation of irrigation volume and nitrogen fertilizer dosage can promote nitrogen flow and conversion in the soil, avoiding the adverse effects of excessive nitrogen on early crop growth. Schulte auf’m Erley et al. (2005) further showed that a reasonable amount of irrigation water enhances nitrogen uptake in quinoa, thereby increasing dry matter accumulation and yield. The experimental results showed that water–nitrogen interactions had a significant threshold effect on dry matter accumulation in quinoa. At low irrigation levels (*W*1), a moderate increase in *N* fertilization could compensate for the effect of water stress on crop growth. However, at medium and high irrigation levels (*W*2, *W*3), excessive *N* application (more than 150 kg/ha) inhibited dry matter accumulation and led to yield reductions of 12.6%–18.9%, consistent with Andersen et al. (2014) and Cui et al. (2020). Excessive nitrogen may inhibit growth and reduce grain quality through mechanisms such as leaching loss (Ma et al., 2023), increased metabolic load on plants (Irin and Hasanuzzaman, 2024), and disruption of carbon–nitrogen balance (Fañanás‐Pueyo et al., 2025). Compared with traditional crops such as corn and wheat (Wang et al., 2024a) (typically with a nitrogen threshold of 180–220 kg/ha), quinoa has a lower nitrogen requirement, reflecting its unique nutrient utilization efficiency and drought-adaptation strategy (Dehghanian et al., 2024). In the conditions of this study, 150 kg/ha was identified as the key threshold for nitrogen fertilizer application in quinoa. Exceeding this range is likely to reduce efficiency and increase environmental risks. Water and nitrogen have been shown to interact positively within an optimal range, but excessive nitrogen application can produce negative effects (Zhang et al., 2008). In this experiment, a dynamic relationship model between quinoa yield, irrigation, and nitrogen application was constructed using regression analysis. The results showed that under medium and high irrigation levels (*W*2, *W*3), when the nitrogen application was less than or equal to 150 kg/ha, additional nitrogen fertilizer significantly increased yield. However, further increases in nitrogen application resulted in minimal yield gains or even a decline, consistent with the law of diminishing returns. The medium water–nitrogen treatment (*W*2*N*3) maximized the fertilizer potential of nitrogen, thereby enhancing quinoa yield. The regression model showed that the highest quinoa yield could reach 4,363.91 kg/ha when the irrigation rate was 2,931.59 m^3^/ha and the nitrogen application rate was 149.24 kg/ha.

Water and nitrogen are two key factors influencing crop yield, and both yield and quality can be significantly affected by their regulation (Correndo et al., 2021). Wang et al. (2024b) showed that, in maize kernels, increased nitrogen fertilizer application not only increased the content of its crude protein and crude fat but also reduced the content of acid-washed fiber. The study by Yin et al. (2025) showed that, within a certain nitrogen fertilizer dosage range, the crude protein content in maize kernels increased with increasing nitrogen fertilizer dosage. The results of the present study are consistent with these findings, as fat, protein, and starch contents in quinoa kernels under the high nitrogen application (*N*4) treatment at the low irrigation level (*W*1) were significantly higher than those in the other nitrogen application treatments. The fat, protein, and starch contents of the kernels under medium and high irrigation levels (*W*2, *W*3) first increased and then decreased with increasing nitrogen application, which is consistent with the findings of Liu et al. (2019), showing that regulating irrigation and nitrogen fertilizer application within a certain range can improve rice yield and kernel quality.

Efficient synergistic utilization of water and nitrogen resources by crops by crops is difficult to achieve through irrigation or nitrogen application alone. The construction of a coupled water–nitrogen management model can enhance crop WUE and nitrogen effectiveness (Isla et al., 2020). Studies have shown that the water–nitrogen ratio is a key indicator of high crop yield and efficiency, and that an appropriate synergistic management strategy can optimize resource efficiency (Liu et al., 2020). This study demonstrates that under low-water conditions (*W*1), WUE increases with rising nitrogen application rates. Under medium to high moisture conditions (*W*2 and *W*3), WUE exhibits a unimodal response to nitrogen application, peaking at the *N*2 level (125 kg/ha). This pattern is closely related to quinoa’s stomatal regulation capacity and drought adaptation strategies: moderate nitrogen supply enhances root water uptake and improves water conversion efficiency, whereas excessive nitrogen may increase stomatal conductance (Matimati et al., 2014) and intensify transpiration (Zhang et al., 2008), thereby reducing water use efficiency. By constructing a regression model to analyze the interaction effect of water and nitrogen, it was found that the synergistic regulation of water and nitrogen use efficiency exhibited a nonlinear parabolic (*R*² > 0.925). The *W*2*N*3 treatment (2,700 m^3^/ha, 150 kg/ha) achieved the optimal synergistic effect of WUE (1.59 g/kg), NAE (26.7 kg/kg), and NPFP (28.66 kg/kg), surpassing the traditional model. The synergistic optimization of WUE, NAE, and NPFP was 23.4% higher than that of the traditional model, confirming the efficiency mechanism of “using water to promote fertilizer” (Li et al., 2015). The results are consistent with the findings that moderate irrigation enhances soil nitrogen effectiveness, agronomic utilization, and nitrogen fertilizer bioproductivity.

Correlation analysis showed that irrigation level was highly significantly negatively correlated (*p* < 0.01) with WUE, indicating that excessive irrigation led to water wastage. Nitrogen application was highly significantly negatively correlated (*p* < 0.01) with yield, dry matter, fat, and protein content, suggesting that excessive nitrogen inhibited yield formation and reduced nitrogen utilization (nitrogen fertilizer bias decreased by 19.5–21.2 kg/kg). Although water–nitrogen interactions had no significant effect on WUE, both WUE and nitrogen efficiency were optimal when applying 125–150 kg/ha of nitrogen under medium irrigation (*W*2). The experimental results indicated that appropriate water–nitrogen ratios enhanced the synergistic utilization of resources, whereas excessive inputs beyond the threshold led to a decreased efficiency, confirming the threshold effect of water–nitrogen coupling and the unique response mechanism of quinoa in the southern border.

## 5 Conclusions

The accumulation of dry matter and nitrogen in quinoa is the main factor determining the grain yield. Inputs of water, nitrogen, and soil nutrients all have a significant impact on quinoa yield. In southern Xinjiang, the recommended water–nitrogen ratio scheme (irrigation volume of 2,700 m^3^/ha and nitrogen application volume of 125–150 kg/ha) can significantly improve the utilization efficiency of water and nitrogen resources, reduce the risk of environmental emissions, and maintain the yield and quality of quinoa. In the current quinoa production in the Xinjiang region, water and nitrogen inputs are generally high (irrigation water often exceeds 3,600 m^3^/ha, and nitrogen application rates range from 180 to 220 kg/ha). The results of this study can provide key theoretical support and a technical basis for promoting water-saving, fertilizer-saving, environmentally friendly, and efficient quinoa production in the region.

## Data Availability

The original contributions presented in the study are included in the article/supplementary material. Further inquiries can be directed to the corresponding authors.
